# Association between bone marrow donor origin and gut microbiota composition following fecal microbiota transplantation in mice

**DOI:** 10.1038/s41598-026-36933-0

**Published:** 2026-03-12

**Authors:** Ryoha Ichimura, Kazuki Tanaka, Isaiah Song, Eisuke Shimizu, Yoko Ogawa, Kazuo Tsubota, Shinji Fukuda

**Affiliations:** 1 Systems Biology Program, Graduate School of Media and Governance, Keio University, Fujisawa, Kanagawa Japan; 2 Institute for Advanced Biosciences, Keio University, Tsuruoka, Yamagata Japan; 3 Gut Environmental Design Group, Kanagawa Institute of Industrial Science and Technology, Kawasaki, Kanagawa Japan; 4 Innovative Microbiome Therapy Research Center, Juntendo University Graduate School of Medicine, Bunkyo-ku, Tokyo, Japan; 5 Department of Ophthalmology, Keio University School of Medicine, Shinjuku-ku, Tokyo, Japan; 6 Transborder Medical Research Center, University of Tsukuba, Tsukuba, Ibaraki Japan

**Keywords:** BMT, FMT, Immune environment, Gut microbiota, Clinical microbiology, Microbial ecology, Microbiome

## Abstract

Fecal microbiota transplantation (FMT) has been reported as a method to directly alter the recipient’s gut microbiota in order to normalize its composition and confer therapeutic benefits. It is essential to maintain a consistent gut microbiota in FMT donors. However, various environmental factors influence the maintenance of gut microbiota composition. We investigated whether bone marrow transplantation (BMT) donor origin, presumably affecting the resultant recipient immune environment, may influence FMT outcomes by conducting a study combining BMT and FMT treatments in mice. Our results show that the origin of the bone marrow donor affected the extent to which the FMT recipient microbiota resembled that of the FMT donor. However, these changes did not reduce the overall microbiota dissimilarity compared to FMT alone. Among the affected microbes, the relative abundance of a mucus-associated bacterium in the family Muribaculaceae was higher, suggesting sensitivity to the combined influence of BMT and FMT donor origins. These findings are consistent with the possibility that bacterial proximity to the intestinal epithelium could modulate persistence patterns in response to distinct post-BMT environments. Altogether, the observed trends suggest that differences associated with bone marrow donor origin may shape post-FMT microbial retention.

**Supplementary Information:**

The online version contains supplementary material available at 10.1038/s41598-026-36933-0.

## Introduction

Currently, various medical treatments are used for diseases related to gut dysbiosis. Among these, fecal microbiota transplantation (FMT) is recognized as one of the most innovative and effective methods due to its ability to alter the recipient gut microbiota. FMT is a procedure to collect feces from a healthy donor and introduce them into a patient’s gastrointestinal tract. This procedure can improve the balance of the recipient’s gut microbiota. Accumulating data indicate that FMT is beneficial for the treatment of *Clostridioides difficile* infection, inflammatory bowel disease, intractable functional constipation, and other gastrointestinal diseases^1–3^. And after FMT, it is imperative that the recipient gut microbiota becomes similar to that of the donor^4^.

The composition of the gut microbiota can be altered due to environmental factors such as diet, probiotics, prebiotics, viruses, drugs (especially antibiotics), genetic factors, and host immunity^5–10^. Therefore, such factors are important to consider when considering the viability of bacteria administered by FMT. As one of these factors, the host immune system also influences the gut microbiota. In turn, symbiotic microorganisms influence the development and function of the immune system, creating a bidirectional relationship between them^10,11^. Consequently, it has been reported that the host’s immune system also influences the degree of similarity between donor and recipient gut microbiota following FMT^12^. Secreted immunoglobulin A (IgA), produced by plasma cells, recognizes specific bacteria and contributes to the regulation of their relative abundance within the gut microbiota^13,14^. IgA has been shown to coat the surfaces of gut commensal bacteria and bind to specific bacteria that naturally coexist in the intestinal mucosa, contributing to the regulation of the relative abundance of host-associated bacteria^13^. It has also been reported that immunocompromised recipients of hematopoietic stem cell transplants and FMT treatments lose their FMT-derived microbiota over time^15^. However, the impact of host immunity on FMT is still unclear in many areas. The development of microbiome-based therapies such as FMT require a deeper understanding of the complex and intricate interactions between the microbiome and the immune system. Therefore, it is necessary to clarify the effects of host immunity on FMT in order to improve donor–recipient similarity of the gut microbiota.

In current studies of the gut microbiota, germ-free mice are usually used as a method to investigate bacteria-host interactions. However, immune functions and physiological conditions in germ-free mice are not appropriate for demonstrating the effects of host immunity on the gut microbiota, as it has been reported that mucosal epithelial barrier function is impaired in germ-free mice, displaying lower antimicrobial peptide production and slower intestinal epithelial cell turnover than in normally reared mice^16^.

Therefore, the purpose of this study is to clarify changes in the gut microbiota during FMT, while also considering the potential influence of BMT donor origin, which may modulate immune environments. BMT involves the transplantation of bone marrow and splenic cells from a donor, and when performed between genetically identical mice (syngeneic BMT), it does not induce graft-versus-host disease and causes only minimal disruption to immune balance^17^. This makes syngeneic BMT a useful tool to induce controlled changes in immune function while maintaining overall immune homeostasis. By considering the environmental conditions of the donor mice used for BMT in combination with microbiota-status of the donors used for FMT, we aimed to evaluate whether differences associated with BMT donor origin are related to patterns of taxon enrichment or persistence of specific bacterial populations during FMT. Conventional (CV) and specific-pathogen-free (SPF) mice have different gut microbiota compositions, with SPF displaying lower microbial diversity^18,19^. Also, mice reared in SPF environments show similar immune profiles to human neonates in that they are characterized by a less-developed immune system compared to CV mice^20^. In summary, we tested the changes in taxon enrichment following FMT in mice with distinct microbiota and bone marrow donor backgrounds by utilizing two different mouse models, CV and SPF, in addition to BMT treatment.

## Method

### Animal experiments

Six-week-old BALB/cAJcl female mice were purchased from CLEA Japan, Inc. (Tokyo, Japan). The mice were kept in a facility with controlled temperatures, humidity, and lighting (12 h light/dark cycle).

**Fig. 1:**
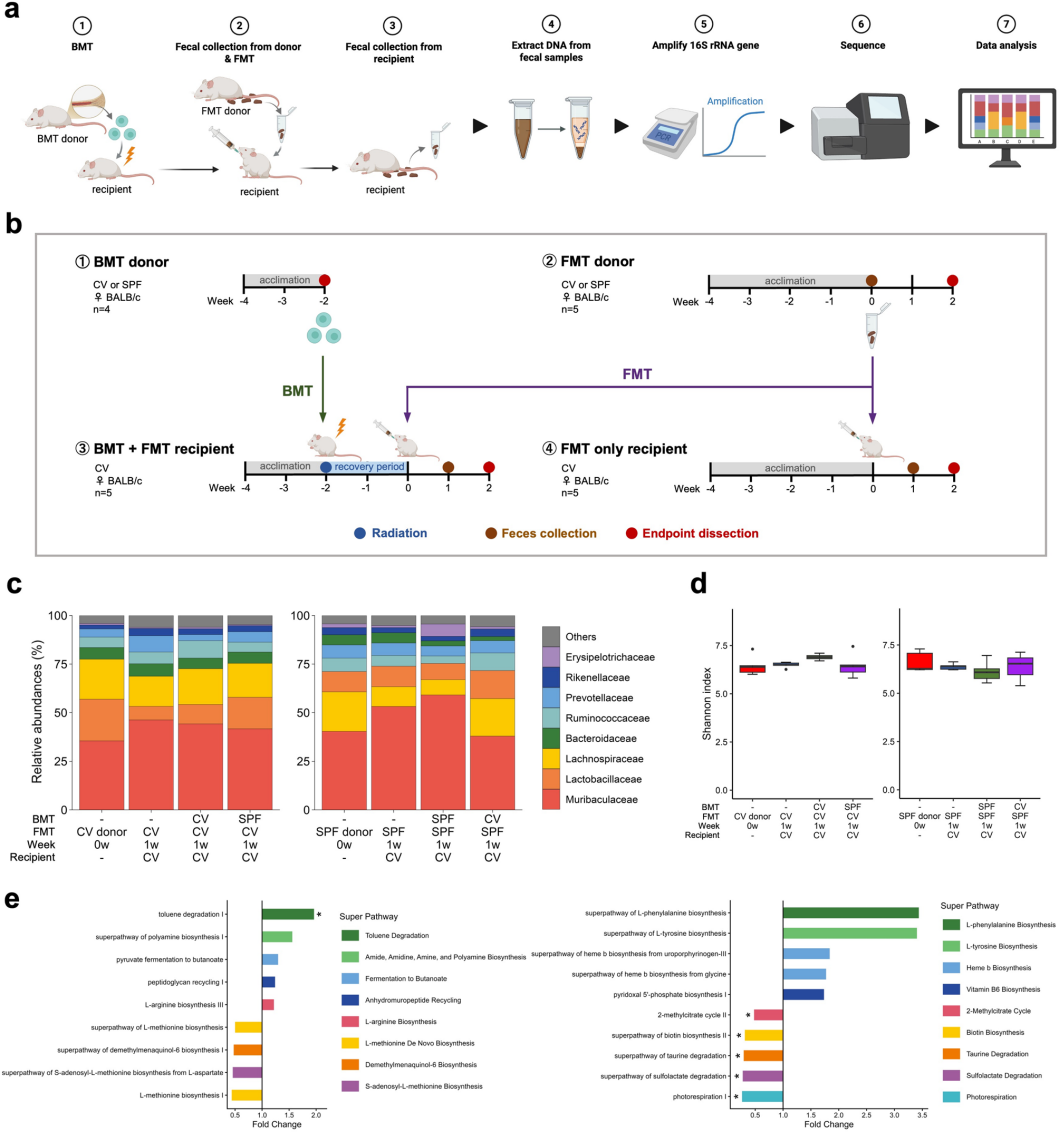
Changes in the gut microbiota caused by BMT and FMT together resulted in significant functional changes. (**a**) Overview of the experimental workflow. (**b**) Experimental schedule. Week numbers are shown relative to the time of FMT (Week 0), and are unified across all groups. Numbers denote the four experimental schedules used in the experiment and correspond to the schedule categories listed in Table 1. (**c**) The composition of the gut microbiota in FMT donor mice at the time of FMT (0w) and of each group one week after FMT (1w) were compared. Average relative abundances of the top-8 most abundant bacterial families in FMT donor mice are shown. (**d**) Comparative results of microbiome alpha diversity between FMT donor mice immediately before FMT (0w) and each transplant implementation group one week after FMT (1w). The Tukey–Kramer test with FWER correction was applied across all pairwise comparisons among four groups (a total of six comparisons). Statistical significance was assessed using adjusted *P* values with a significance threshold of *P* < 0.05. Based on the test results, no significant differences were found between groups for either of these parameters. (**e**) Changes in estimated pathway abundance by BMT when receiving FMT. Comparisons are separated based on the fecal source used for FMT (CV, left; SPF, right). Significant differences based on Mann–Whitney U test are indicated by * *P* < 0.05. This non-parametric test yields unadjusted *P* values. Pathways were selected for visualization by ranking pathway-level comparisons based on *P* values, and the 10 pathways with the lowest *P* values in each comparison (five with increased and five with decreased predicted abundance) were retained. No filtering based on fold-change thresholds was applied.

We established ten experimental groups based on different experimental schedules (Fig. 1a and b). BMT donor mice (*n* = 4) were purchased at six weeks of age and housed under either CV or SPF conditions for approximately two weeks after arrival to allow acclimatization before BMT. “SPF” refers to animals maintained in barrier facilities with strict pathogen exclusion, while “CV” refers to animals housed in non-barrier, open cages without pathogen exclusion, which may include common murine commensals. Bone marrow and spleen were collected from these BMT donors on the day of BMT. FMT donor mice (*n* = 5) were purchased at six weeks of age and maintained under their respective CV or SPF conditions for four weeks after arrival to allow acclimatization before FMT. Fecal samples were collected from these FMT donors immediately before FMT. Recipient mice were purchased at six weeks of age and housed under CV conditions throughout the experimental period. The BMT + FMT recipient group (*n* = 5) was acclimatized for approximately two weeks after arrival and then received both bone marrow and spleen cells from BMT donors. Following a two-week recovery period, these mice received FMT using feces from FMT donors. In contrast, the FMT-only recipient group (*n* = 5) was acclimatized for approximately four weeks under CV conditions before receiving FMT, without prior BMT. Fecal samples were collected from recipient mice one week after FMT. After 4 weeks of BMT, the animals were dissected, and cecum and colon were collected and stored at −80˚C until further analysis. Donor characteristics for each experimental group are shown in Table 1, and the experimental schedule is illustrated in Fig. 1a and b. Mice within the same experimental group were co-housed throughout the experimental period. This group-based housing strategy was implemented to minimize inter-individual variation in gut microbiota composition and to ensure uniform microbial exposure under consistent experimental conditions. All animals were euthanized with deep isoflurane anesthesia and cervical dislocation. Animal experiments were approved by the Institutional Animal Care and Use Committee of Keio University (09152 and A2022-178). All methods were carried out in accordance with relevant guidelines and regulations, and the study was conducted in accordance with institutional regulations and the ARRIVE guidelines. All efforts were made to minimize suffering.

**Table 1:** List of experimental groups.

Group	Designation	BMT donor	FMT donor	Schedule^1^
CV BMT donor	Donor	-	-	①
CV FMT donor^2^	Donor	-	-	②
SPF BMT donor	Donor	-	-	①
SPF FMT donor^3^	Donor	-	-	②
CV_FMT	Recipient	-	CV	④
CV_BMT + CV_FMT	Recipient	CV	CV	③
SPF_BMT + CV_FMT	Recipient	SPF	CV	③
SPF_FMT	Recipient	-	SPF	④
CV_BMT + SPF_FMT	Recipient	CV	SPF	③
SPF_BMT + SPF_FMT	Recipient	SPF	SPF	③

^1^Schedule numbers (①–④) correspond to the experimental timelines shown in Fig. 1b.

^2^Also referred to as CV_donor in the data.

^3^Also referred to as SPF_donor in the data.

### Bone marrow transplantation

BMT was performed as described previously^21^. The tibia and femur were harvested from donor mice under aseptic conditions and washed with PBS (15 mL × 3 times). The bones were then finely crushed, 10 mL of RPMI 1640 (GIBCO) were added, and the supernatant was passed through a 100-µm filter. The supernatant was collected and 5 mL of RPMI was added.

Spleens were also collected from donor mice under aseptic conditions and were passed through a 100-µm filter. They were then mixed with 10 mL of RPMI after light crushing, and the suspension was passed through a 100-µm filter again. The filtrate was centrifuged at 1,200 rpm for 10 min at 4˚C and the supernatant was collected. To the supernatant, 5 mL of ACK KYSING BUFFER (QUALITY BIOLOGICAL, INC. 118–156-721) was added, stirred, and filtered again under the same conditions. The supernatant was collected and 5 mL of RPMI was added. Finally, we adjusted bone marrow cells to 1 × 10^7^ cells/mL and spleen cells to 2 × 10^7^ cells/mL, respectively.

After recipient mice were lethally irradiated with a single fraction of 7 Gy (150 kV, 10 mA, 430 nm), they were injected by tail vein with female donor bone marrow (Approx. 1 × 10^6^/mouse) and spleen (Approx. 2 × 10^6^/mouse) cells suspended in RPMI 1640 within 24 h after irradiation. Transplanted animals were maintained in sterile MicroIsolator cages and supplied with autoclaved food and acidified water.

### Fecal microbiota transplantation

For FMT administration, one fresh stool sample was collected from each mouse in the FMT donor group. A total of 100 mg of the pooled feces were immediately placed into 1 mL of sterile saline and were suspended in a homogenizer. Then, the dissolved feces were spun down using a benchtop microcentrifuge for a few seconds at room temperature to precipitate large debris. The supernatant was collected and 300 µL of bacterial suspension were then delivered to each recipient mouse via oral gavage within 15 min.

### DNA extraction and 16 S rRNA gene sequencing

Each stool sample was snap-frozen with liquid nitrogen following collection and was stored in a sterile container within a −80˚C refrigerator until use. Fecal DNA isolation was performed as described previously with some modifications^22^. In short, fecal samples were lyophilized for approximately 18 h using a VD-800R lyophilizer (TAITEC, Nagoya, Aichi, Japan). Each freeze-dried fecal sample was subjected to vigorous shaking (1,500 rpm. for 10 min) using a Shake Master (Biomedical Science, Shinjuku, Tokyo, Japan) combined with four 3.0-mm zirconia beads. Ten mg of each fecal sample was mixed with approximately 100 mg of 0.1-mm zirconia/silica beads, 300 µL DNA extraction buffer (TE containing 1% (w/v) sodium dodecyl sulfate), and 300 µL of phenol/chloroform/isoamyl alcohol (25:24:1) and was subjected to vigorous shaking (1,500 rpm. for 5 min) using a Shake Master (Biomedical Science, Shinjuku, Tokyo, Japan). The resulting emulsion was subjected to centrifugation at 12,000 rpm for 10 min at room temperature, and bacterial genomic DNA was purified from the aqueous phase by a standard phenol/chloroform/isoamyl alcohol protocol. RNA was removed from the sample by RNase A treatment; the resulting DNA sample then was purified again by another round of phenol/chloroform/isoamyl alcohol treatment. The concentration of gene DNA was measured using the Nanodrop 2000 (Thermo Scientific, USA). DNA samples were stored at − 30˚C until all samples were ready for sequencing.

16 S rRNA genes in the fecal DNA samples were analyzed using a MiSeq sequencer (Illumina). The V1-V2 region of the 16 S rRNA gene was amplified from the fecal DNA (approximately 10 ng per reaction) using a bacterial universal primer set consisting of primers 27Fmod with an overhang adapter (5′-ACACTCTTTCCCTACACGACGCTCTTCCGATCTAGRGTTTGATYMTGGCTCAG-3′) and 338R with an overhang adapter (5′-GTGACTGGAGTTCAGACGTGTGCTCTTCCGATCTTGCTGCCTCCCGTAGGAGT-3′)^23,24^. PCR was performed with Tks Gflex DNA Polymerase (Takara Bio, Inc., Kusatsu, Shiga, Japan) and amplification via the following program: one cycle of denaturation at 98˚C for 1 min; 20 cycles of amplification at 98˚C for 10 Sect. 55˚C for 15 s, and 68˚C for 30 s; and a final extension at 68˚C for 3 min. Amplicons were confirmed to be amplified using the QIAxcel DNA Screening Kit (QIAGEN, 929004) and QIAxcel Advanced System (QIAGEN, 9002123). The amplified products were purified using Agencourt AMPure XP kits (Beckman Coulter, Atlanta, GA, USA). The purified products were then further amplified using the following primer pair: a forward primer (5′- AATGATACGGCGACCACCGAGATCTACAC-NNNNNNNN-ACACTCTTTCCCTACACGACGC-3′) containing the P5 sequence, a unique 8-bp barcode sequence for each sample (indicated by the string of Ns), and an overhang adapter; and a reverse primer (5′-CAAGCAGAAGACGGCATACGAGAT-NNNNNNNN-GTGACTGGAGTTCAGACGTGTG-3′) containing the P7 sequence, a unique 8-bp barcode sequence for each sample (indicated by the string of Ns), and an overhang adapter. After purification using Agencourt AMPure XP kits, the purified products were mixed in approximately equal molar concentrations to generate a 4 nM library pool, after which the final library pool was diluted to 6 pM, including a 10% PhiX Control v3 (Illumina) spike-in for sequencing. Finally, MiSeq sequencing was performed according to the manufacturer’s instructions. In this study, 2 × 300-bp paired-end sequencing was employed.

### Analysis of 16 S rRNA gene sequencing data

Analysis of 16 S rRNA gene sequences was performed as described previously with some modifications^25^. Reads were processed using the Quantitative Insights into Microbial Ecology (QIIME 2)^26^. Demultiplexed reads were processed using QIIME2 with DADA2-based denoising procedures^27^. Amplicon sequence variants (ASV) were assigned based on 99% sequence similarity according to SILVA 132. Alpha diversity was assessed using Shannon as determined by QIIME 2, while beta diversity (species complexity) was assessed by principal coordinates analysis (PCoA) and cluster analysis using QIIME 2. To statistically evaluate differences in overall microbial community composition, permutational multivariate analysis of variance (PERMANOVA) was performed using the adonis2 function in the vegan package in R. PERMANOVA is a distance-based, permutation-derived method that evaluates group-level differences in multivariate community structure by partitioning variation in a dissimilarity space^28^; therefore, *P* values were not adjusted for multiple comparisons. Analyses were conducted based on unweighted and weighted UniFrac distance matrices, with all parameters set to their default values unless otherwise specified. Statistical significance was assessed using permutation-based *P* values derived from the pseudo-F statistic, with significance defined as *P* < 0.05, and effect sizes were expressed as R² values. PERMANOVA analyses were performed separately according to the FMT donor source (CV or SPF), using the same sample groupings as those shown in the PCoA plots. Homogeneity of multivariate dispersion was assessed using the betadisper function in the vegan package in R. Also, we determined our samples’ genetic and functional content by utilizing the Phylogenetic Investigation of Communities by Reconstruction of Unobserved States (PICRUSt) v2.3.0 b software and filtering the feature table and “representative sequences (rep-seqs.qza file)” using the QIIME2 pipeline^30^. Pathway-level comparisons based on PICRUSt2 predictions were performed in an exploratory manner to identify putative functional trends. Briefly, rep-seqs.qza was converted into FASTA format; these 16 S sequence variants were then aligned and mapped to a maximum-likelihood-based phylogenetic tree. Then, PICRUSt2 allowed the prediction of the 16 S gene content and gene families content for each mapped sequence on the tree. In the final step of the PICRUSt2 pipeline, two tables of predicted counts were produced: the former was relative to the per-sample enzyme content, while the latter showed the inferred pathway abundance per sample, named by their MetaCyc v24.5 Identifiers^31^. For pathway-level statistical comparisons, the Mann–Whitney U test was used to compare two groups, yielding unadjusted *P* values, with statistical significance defined as *P* < 0.05. For visualization, pathways were ranked based on the *P* values obtained from pathway-level comparisons, and the 10 pathways with the lowest *P* values in each comparison (five with increased and five with decreased predicted abundance) were selected. No correction for multiple comparisons or filtering based on fold change thresholds was applied. Differences in ASV abundance between groups were identified using LEfSe v1.1.2, which was implemented within the QIIME2 environment^29^. LEfSe integrates the non-parametric Kruskal–Wallis test and considers taxa with unadjusted *P* values < 0.05. In addition to this threshold, a logarithmic LDA score cutoff of > 3.2 was applied to identify taxa with significant effect sizes. Because LEfSe is intended as an exploratory feature-selection approach, taxa identified by LEfSe were treated as candidate features and were subsequently evaluated using targeted statistical tests with appropriate multiple-comparison control, where applicable. The full command logs used for QIIME2, PICRUSt2, and LEfSe analyses are available at https://github.com/keio-ichimura/16S_scripts.

### Statistical analysis

Data are presented as mean ± standard error of the mean. Statistical analyses were conducted based on the specific comparison objectives. Mann–Whitney U test was used to compare two groups. This non-parametric test yields unadjusted *P* values. For multiple comparisons, the Tukey–Kramer test was applied when comparing all pairs of groups, while Dunnett’s multiple comparison test was used when comparing multiple groups against a control group. Both the Tukey–Kramer and Dunnett tests incorporate family-wise error rate (FWER) control by design, and the corresponding *P* values were adjusted accordingly. Statistical significance was defined as *P* < 0.05. Statistical tests were performed using the multcomp package v1.4.23 in R version 4.2.3 (R Core Team, 2023).

## Results

### Changes in the gut microbiota caused by the combination of BMT and FMT resulted in significant functional changes

We conducted BMT and FMT combination studies using donor mice reared in either CV or SPF environments and recipient mice reared in a CV environment only (Fig. 1a and b). The experimental groups are shown in Table 1 and can be broadly divided into CV/SPF bone marrow and feces donors, and recipients of feces and/or bone marrow from said CV and/or SPF donors.

The taxonomic diversity was profiled by sequencing the 16 S ribosomal RNA gene of the gut microbiota using fecal samples from each group of recipient mice one week after FMT (Fig. 1c). Notably, one mouse in the CV_BMT + CV_FMT group and two mice in the CV_BMT + SPF_FMT group died within one week after FMT; therefore, fecal samples at one week post-FMT were not obtained from these animals. Differences in the gut microbiota between CV and SPF donor mice used for FMT were compared using LEfSe analysis. The results showed that taxa belonging to the class Coriobacteriia within the phylum Actinobacteria, particularly the genus *Enterorhabdus*, were enriched in CV donor mice. In contrast, *Bacteroides massiliensis* dnLKV3 was more abundant in SPF donor mice (Supplementary Fig. 1).

When we compared the microbiome alpha diversity of recipient mice to assess the effects of differences in BMT donor origin on microbial diversity, no significant differences in diversity were detected (Fig. 1d).

In order to clarify not only the gut microbiota community structure but also the functional changes that may have resulted from changes in the gut microbiota as a result of the combination of BMT and FMT, we used PICRUSt2 software to predict functional potential by comparing the inferred pathways in relation to BMT donors based on the source of the feces used to seed the FMT recipients (CV or SPF mice). PICRUSt2 is a software for predicting functional abundances based on marker gene sequences. Analyzing the pathway abundances per sample as determined using PICRUSt2, the 10 pathways that showed the lowest *P* values in each comparison (five with increased predicted abundance and five with decreased predicted abundance) were selected. These predicted pathways are shown as fold-changes in mice that received feces and bone marrow from donor groups composed of multiple individuals maintained under the same microbiota-status (i.e., donors originating from the same husbandry category, either CV or SPF), compared to those from different microbiota-status donor mice. The grouping was determined based on the source of the feces used to seed the FMT recipients (CV or SPF mice) (Fig. 1e). Notably, in the CV_BMT + CV_FMT group, pathways related to amino acid biosynthesis such as arginine and polyamine synthesis showed higher predicted abundances. In the SPF_BMT + SPF_FMT group, pathways involved in aromatic amino acid metabolism including tyrosine and phenylalanine showed higher predicted levels. These results suggest that putative metabolic pathways for amino acid biosynthesis and utilization were predicted to be influenced when bone marrow and feces were transplanted from the same microbiota-status donor mice. However, as these functional differences were inferred solely from PICRUSt2 predictions without metagenomic or metabolomic validation, they should be verified through future multi-omics analyses.

### Differences in BMT donor origin affect post-FMT microbiota composition

Since the previous results showed that the combination of BMT and FMT resulted in changes in bacterial community function, we next examined what bacteria were affected by BMT specifically.

**Fig. 2:**
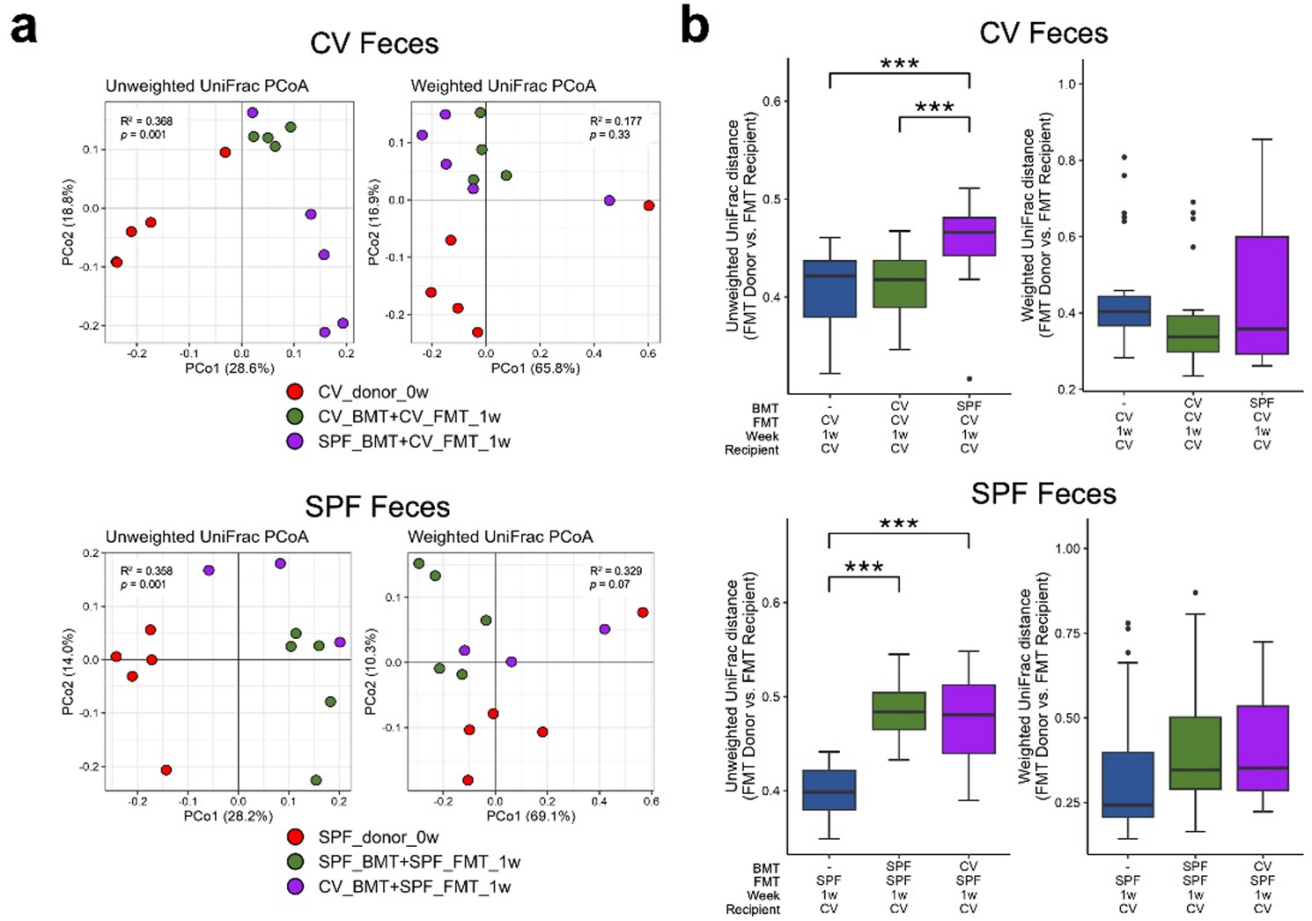
Differences in BMT donor origin affect post-FMT microbiota composition. (**a**) Changes in gut microbiota composition after BMT and FMT treatment. Gut microbiota profiles of FMT donors at the time of FMT (0w) and BMT + FMT recipient groups one week after FMT (1w) were compared. PCoA plots are based on unweighted (qualitative) and weighted (quantitative) UniFrac distances and are separated by FMT fecal source. Statistical support for differences in community composition was assessed by PERMANOVA, a permutation-based global test of community-level differences; therefore, *P* values were not adjusted for multiple comparisons. The results of PERMANOVA are shown within each panel as R² values and *P* values. Full PERMANOVA results are summarized in Supplementary Table 1. (**b**) Unweighted and weighted UniFrac measures between FMT donor mice at the time of FMT (0w) and their associated FMT recipient mice at one week after FMT. Error bars indicate standard deviation. The Tukey–Kramer test with FWER correction was applied across all pairwise comparisons among three groups (a total of three comparisons), and statistical significance was assessed using adjusted *P* values, with significant differences indicated by *** *P* < 0.001.

First, PCoA based on UniFrac distances was conducted in order to clarify differences in microbiota composition as a result of BMT, and groups were separated based on the source of the feces used to inoculate the FMT recipients (CV or SPF donor mice). The analysis was performed using both unweighted and weighted UniFrac distance metrics. Distinct microbiota compositions were observable between treatment groups in unweighted UniFrac PCoA compared to weighted UniFrac PCoA (Fig. 2a). These visual patterns were supported by PERMANOVA analyses (Supplementary Table 1). When analyses were stratified by the source of the feces used to inoculate the FMT recipients (CV or SPF donor mice), no significant differences in multivariate dispersion were detected among groups for either unweighted or weighted UniFrac distances. Under these conditions, PERMANOVA based on unweighted UniFrac distances revealed significant differences in community composition between groups (CV: R² = 0.368, *P* = 0.001; SPF: R² = 0.358, *P* = 0.001). In contrast, PERMANOVA based on weighted UniFrac distances did not show statistically significant differences (CV: R² = 0.177, *P* = 0.330; SPF: R² = 0.329, *P* = 0.070).

We also compared the dissimilarity between each FMT-recipient microbiota and its associated donor microbiota by comparing the unweighted and weighted UniFrac measures between groups of FMT donor mice at the time of FMT (0w) and recipient mice at one week after FMT (1w) to evaluate the similarity of gut microbiota composition (Fig. 2b). The results showed no significant differences between groups in the weighted analysis, but significant differences were found in the unweighted analysis. Also, after FMT using CV mouse donor feces, the microbiota dissimilarity between donors and recipients was significantly higher in the SPF_BMT + CV_FMT group compared to the CV_BMT + CV_FMT group. In contrast, the microbiota dissimilarity between donors and recipients did not change with FMT using SPF mouse donor feces, regardless of bone marrow donor origin. These results suggest that the gut environment of the bone marrow donor exerts a greater effect on the degree to which donor-derived microbial signatures are reflected in the recipient microbiota when feces are sourced from CV mice rather than SPF mice.

### BMT and FMT from the same microbiota-status donor mouse significantly increased the relative abundances of bacteria from the family muribaculaceae

**Fig. 3:**
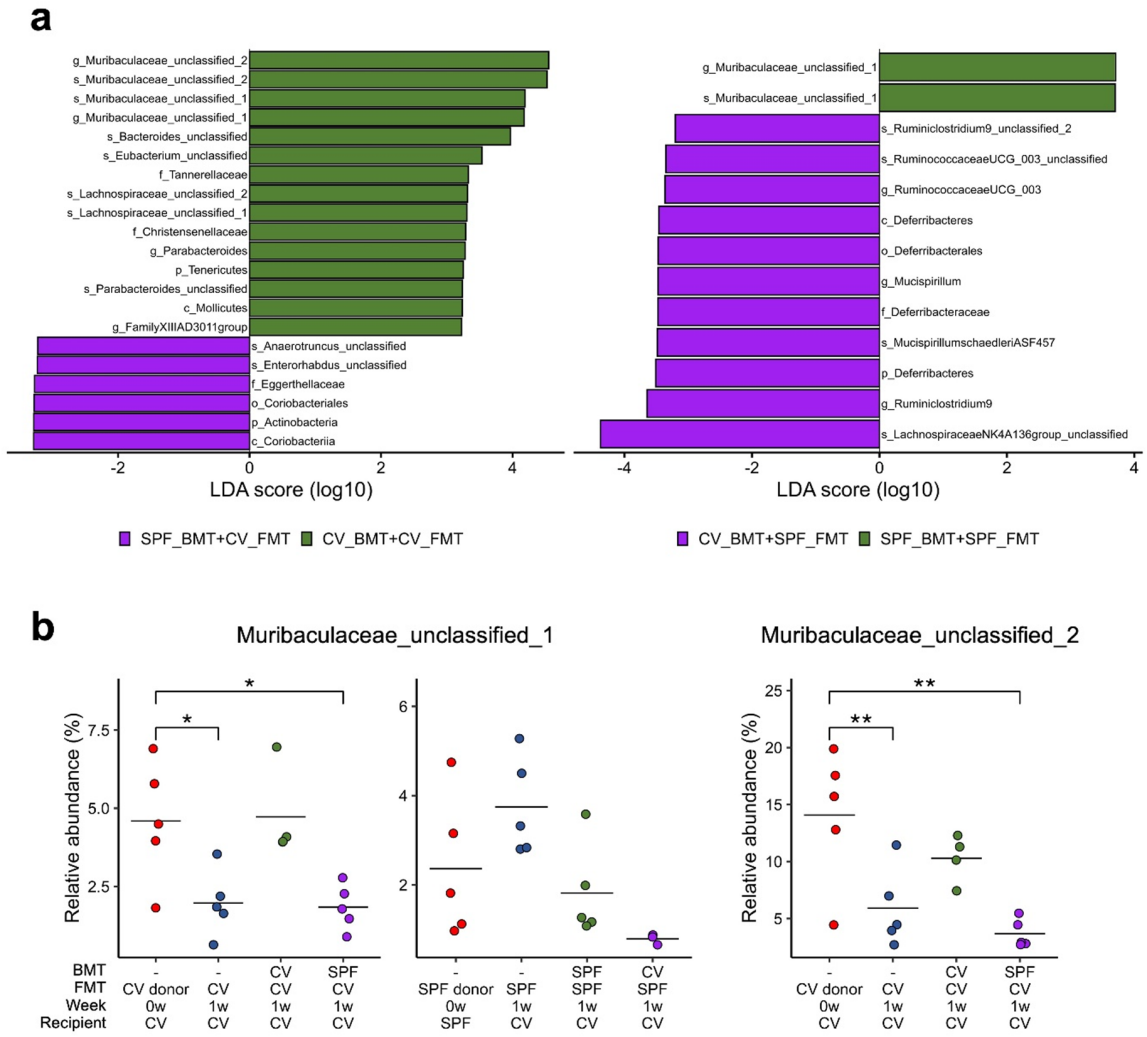
BMT and FMT from the same microbiota-status donor mouse significantly increased relative abundance of Muribaculaceae. (**a**) LEfSe analysis results. Comparisons are separated based on the fecal source for FMT treatment (CV, left; SPF, right). Taxa were identified using LEfSe with a Kruskal–Wallis test (unadjusted *P* < 0.05) and a logarithmic LDA score cutoff > 3.2. (**b**) Relative abundance comparisons of two Muribaculaceae-associated ASVs. Dunnett’s test with built-in FWER correction was applied to three comparisons versus the control group. Statistical significance was assessed using adjusted *P* values, with significant differences indicated by * *P* < 0.05, ** *P* < 0.01.

We conducted LEfSe to identify bacteria that were differentially associated with differences in BMT donor origin, and compared the identified bacteria relative to BMT donors based on the source of the feces used to seed the FMT recipients (CV or SPF mice) (Fig. 3a). Results showed that bacteria from the family Muribaculaceae were specifically affected when bone marrow and feces were transplanted from the same microbiota-status donor mice. Others included members of the class Coriobacteriia in the SPF_BMT + CV_FMT group, and the phylum Deferribacteres and the family Ruminococcaceae in the CV_BMT + SPF_FMT group.

We compared the relative abundances of the Muribaculaceae between FMT donor mice at the time of FMT (0w) and 1 week later (1w) to evaluate changes in their representation after FMT. These bacteria were notably affected, as shown in Fig. 3a. We found that certain Muribaculaceae increased when BMT and FMT were performed using the same microbiota-status donor mice. A comparison of their relative abundances revealed that levels were significantly higher when BMT and FMT were performed using donor mice with the same microbiota-status than when those of different microbiota-status were used. In such cases, the recipient microbiota composition became more similar to that of the FMT donors (Fig. 3b).

## Discussion

In this study, we examined how the gut microbiota of recipient mice changed when FMT was performed following BMT. When we focused on specific gut bacterial species by comparative analysis of the gut microbiota between groups, we found that the donor origin of the bone marrow used for BMT during FMT was associated with increased abundances of mucus-associated bacteria presumably living in the vicinity of the intestinal mucosa.

Comparative analysis of the gut microbiota between groups and searching for bacterial biomarkers showed significant differences in some bacteria. Among them, comparative analysis of the relative abundance of Muribaculaceae in donors and in each group showed that their levels were significantly higher with the combination of BMT and FMT from the same microbiota-status donor mice compared to those of different microbiota-statuses. Under this condition, the recipient microbiota composition became more similar to that of the FMT donors.

**Fig. 4:**
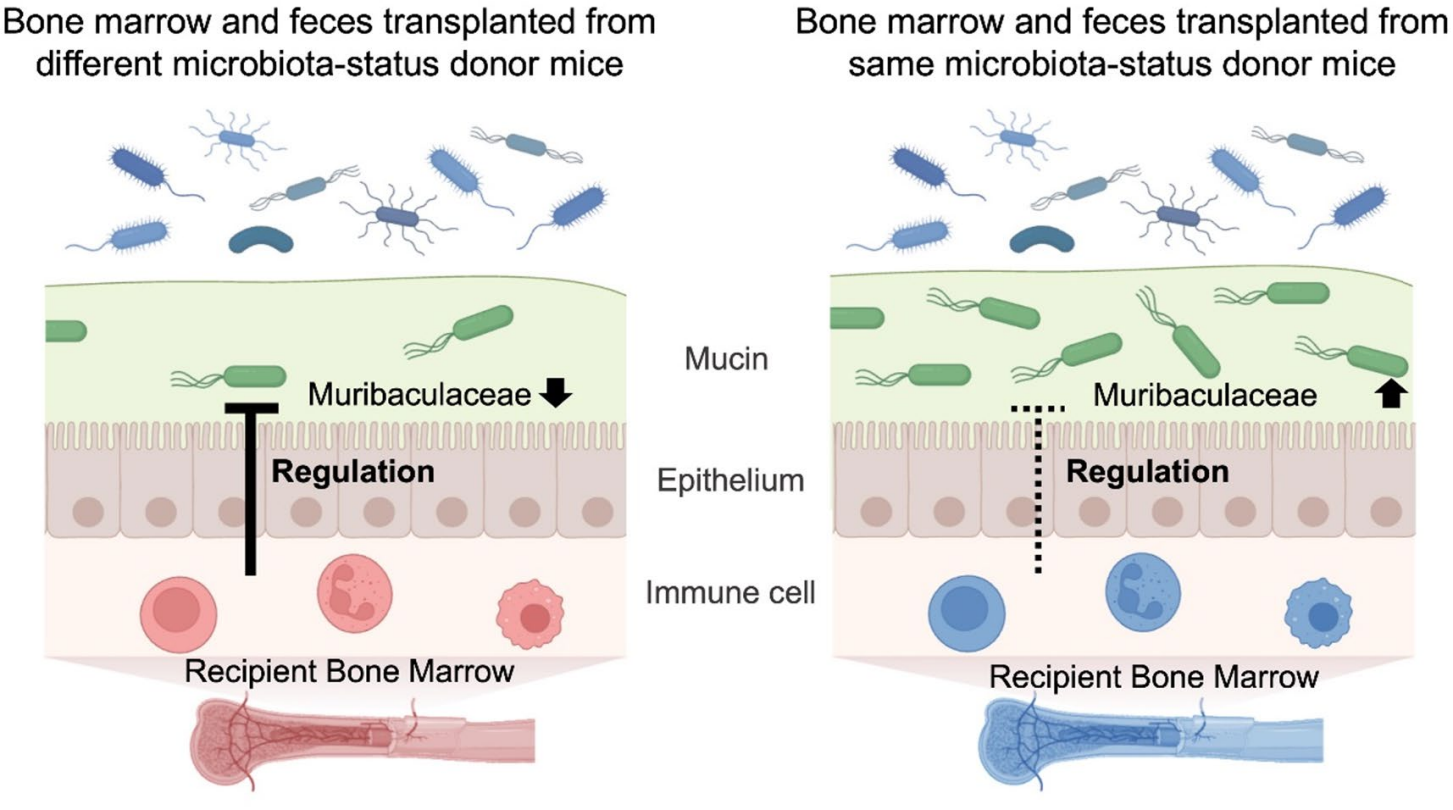
Hypothetical mechanism of Muribaculaceae increase by combination BMT and FMT. Left panel shows transplantation of bone marrow and stool from the same microbiota-status donor mouse; right panel shows transplantation of bone marrow and stool from a different microbiota-status donor mouse.

According to these findings, when both the BMT donor and FMT donor originate from the same housing environment category and reflect a consistent combination of microbiota status and immunological background in the treatment recipient, the resulting conditions may permit an increase in the relative abundance of Muribaculaceae, which could help maintain their levels following BMT and FMT. Conversely, when they come from different housing environments with divergent microbiota compositions and bone marrow characteristics, the conditions may not support this increase in Muribaculaceae abundance, resulting in a lower presence of these bacteria even when BMT is performed before FMT. Muribaculaceae are mucus-associated bacteria that live in the vicinity of the intestinal mucosa^32,33^. Symbiosis between the host and the gut microbiota is dictated by the physical location of these organisms along the gastrointestinal tract, including their proximity to the gut epithelium^34,35^. Taken together, these findings suggest the possibility that physiological effects arising from donor-derived BMT, likely including changes in the immune environment, may promote taxon enrichment among gut microbes that occupy mucus-associated niches when FMT is performed (Fig. 4).

Immune-related factors such as IgA responses have been reported to affect mucus-associated taxa in general^34^. However, this study did not include direct immunological assessments such as IgA binding assays or immune cell profiling and did not evaluate whether differences in donor immune status (e.g., CV vs. SPF) affected immune reconstitution following BMT. Accordingly, interpretations regarding immune-related effects of BMT are speculative. Additionally, as the findings are based on a single experimental trial, further validation in reproducible experiments under controlled conditions is necessary. Previous studies have shown that gut microbiota composition can vary markedly even among genetically identical or related inbred mice. Vendor source, research institution, room-level environment, cage conditions, and husbandry protocols have collectively been identified as environmental and facility-dependent factors that strongly influence microbial profiles and host metabolism^36,37^. These factors may therefore limit the generalizability and reproducibility of our findings.

UniFrac analysis of donor mice used for FMT and of each group one week after FMT showed that differences in BMT donor origin at the time of FMT did not reduce the overall microbiota dissimilarity compared to FMT only. We speculate that this is due to the effects of radiotherapy prior to BMT. To achieve immune ablation, BMT is preceded by conditioning regimens such as total body irradiation (TBI) and/or the administration of chemotherapeutic agents, which have been reported to induce side effects^38^. TBI not only ablates bone marrow cells but may also cause acute damage to the gastrointestinal tract. This damage promotes the leakage of bacterial components from the gut and progression into systemic inflammation^39^. In this manner, BMT treatment affects the host microbiota^40^. One limitation of this study is the absence of a BMT-only or sham-FMT control group. Consequently, we cannot fully exclude the possibility that the observed gut microbiota profiles are at least partially attributable to irradiation or other conditioning regimens. Furthermore, the lack of a sham-FMT group limits our ability to distinguish the biological effects of FMT from procedural influences associated with its administration. Future studies incorporating such control groups will be essential to disentangle the relative contributions of conditioning regimens, FMT, and procedural artifacts. Therefore, further research is needed on ways to improve FMT in these situations.

## Supplementary Information

Below is the link to the electronic supplementary material.

Supplementary Material 1

## Data Availability

The datasets generated and/or analysed during the current study are available in the DNA Data Bank of Japan (DDBJ) repository under accession number PRJDB19872.
