# Supplementary material for: Association between bone marrow donor origin and gut microbiota composition following fecal microbiota transplantation in mice
Source: Sci Rep. 2026 Mar 12;16:13314. doi: 10.1038/s41598-026-36933-0 (PMC13106783; doi:10.1038/s41598-026-36933-0)
Supplement: Supplementary file 1 — Supplementary Material 1 [file 41598_2026_36933_MOESM1_ESM.pdf]

Association between bone marrow donor origin and gut microbiota composition following fecal microbiota transplantation in mice

Ryoha Ichimura[1-3], Kazuki Tanaka[1-4], Isaiah Song[2], Eisuke Shimizu[5], Yoko Ogawa[5], Kazuo Tsubota[5], Shinji Fukuda[1-4,6,*]

1 Systems Biology Program, Graduate School of Media and Governance, Keio University, Fujisawa, Kanagawa, Japan
2 Institute for Advanced Biosciences, Keio University, Tsuruoka, Yamagata, Japan
3 Gut Environmental Design Group, Kanagawa Institute of Industrial Science and Technology, Kawasaki, Kanagawa, Japan
4 Innovative Microbiome Therapy Research Center, Juntendo University Graduate School of Medicine, Tokyo, Japan
5 Department of Ophthalmology, Keio University School of Medicine, Tokyo, Japan
6 Transborder Medical Research Center, University of Tsukuba, Tsukuba, Ibaraki, Japan

*Correspondence:
Prof. Shinji Fukuda,
sfukuda@sfc.keio.ac.jp

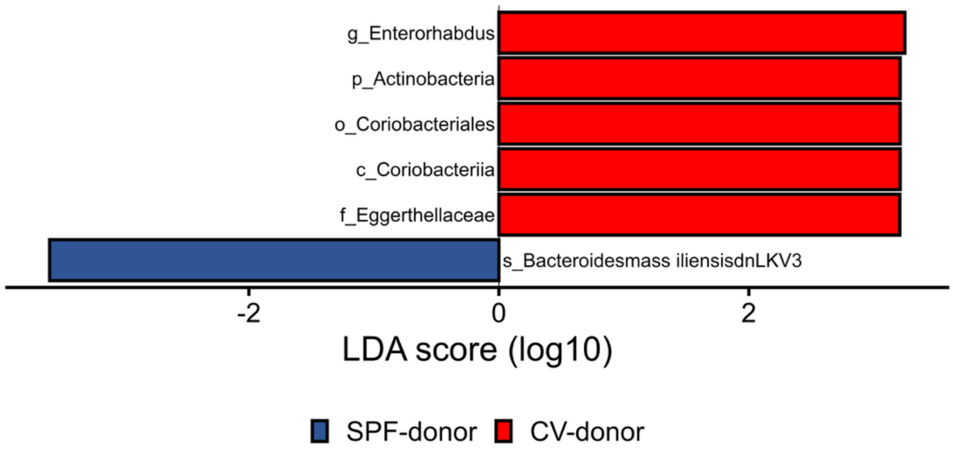

Supplementary Fig. 1: Differences in the gut microbiota between CV and SPF donor mice used for FMT.

LEfSe analysis results showing bacterial taxa that explain differences in the gut microbiota between CV and SPF donor mice used for FMT. The larger the LDA score, the more likely the taxon accounts for differences in the group.

Supplementary Table 1

FMT donor	Distance metric	Pseudo-F	R^2	p value
CV	Unweighted UniFrac	3.21	0.368	0.001
CV	Weighted UniFrac	1.18	0.177	0.330
SPF	Unweighted UniFrac	2.79	0.358	0.001
SPF	Weighted UniFrac	2.45	0.329	0.070

Supplementary Table 1: PERMANOVA results based on UniFrac distances according to FMT donor. Differences in fecal microbiota composition were assessed using PERMANOVA implemented with the adonis2 function, based on unweighted and weighted UniFrac distance matrices. Pseudo-F values, effect sizes (R^2), and permutation-based *P* values are shown. PERMANOVA provides a permutation-based global test of community-level differences; therefore, *P* values were not adjusted for multiple comparisons. The "FMT donor" column corresponds to the FMT donor origin, defined in the same manner as the "FMT donor" column in Table 1. For the CV FMT condition, comparisons included fecal microbiota from CV FMT donor mice at the time of FMT (0w) and from recipient mice one week after FMT (1w), the latter including the CV_BMT+CV_FMT and SPF_BMT+CV_FMT groups. For the SPF FMT condition, comparisons included fecal microbiota from SPF FMT donor mice at the time of FMT (0w) and from recipient mice one week after FMT (1w), the latter including the SPF_BMT+SPF_FMT and CV_BMT+SPF_FMT groups. These groups are identical to those used in Figure 2a.
